# Healthy Food Voucher Programs: Global Evidence on Structure, Implementation, and Nutrition-Related Outcomes

**DOI:** 10.1016/j.advnut.2025.100530

**Published:** 2025-10-01

**Authors:** Jonathan Lara-Arevalo, Camila Corvalan, Isabel Pemjean, Daniela Montes de Oca, Shu Wen Ng, Lindsey Smith Taillie

**Affiliations:** 1 Department of Nutrition, Gillings School of Global Public Health, University of North Carolina at Chapel Hill, Chapel Hill, North Carolina, United States; 2 CIAPEC, Institute of Nutrition and Food Technology, University of Chile, Macul, Chile; 3 Carolina Population Center, University of North Carolina at Chapel Hill, Chapel Hill, North Carolina, United States

**Keywords:** healthy food subsidies, food assistance programs, food vouchers, food incentives, program structure, food access, dietary quality, low-income, nutritional outcomes, food security

## Abstract

Healthy food voucher programs (HFVPs) provide lower-income participants with benefits to purchase healthy, nutrient-dense foods and are a promising strategy for improving dietary and nutritional outcomes. HFVPs can complement policies aimed at reducing unhealthy food consumption, contributing to improved food security, dietary outcomes, and reducing nutritional disparities. Understanding the structural factors that make these programs acceptable and effective in improving dietary patterns is essential for designing impactful HFVPs. However, updated evidence on these components is limited. This narrative review focuses on incentive programs that provide voucher benefits for healthy foods, synthesizing global evidence on program structure components (i.e., participant eligibility and enrollment, benefit delivery and timing, eligible products, benefit value, program duration, retail venues, and inclusion of nutrition education) that may influence program impact. It also summarizes diet and nutrition-related outcomes by country’s income level, when possible. Key determinants of program acceptability included positive interactions with program and retail staff, available multilingual information, electronic benefits over physical ones, a variety of eligible healthy foods, and including local markets as participating venues. Additionally, offering remote enrollment options, using mail delivery or electronic benefits to avoid transportation costs, adjusting benefits for inflation and household size, allowing redemption in various retail venues, and coupling benefits with engaging nutrition education activities were factors influencing program effectiveness. Most evidence indicates that HFVPs increase the purchase and consumption of healthy foods, improve food security, and enhance nutrition knowledge. However, mixed results were found regarding diet quality indicators, physical health outcomes, and mental health. Factors such as insufficient benefit size, inflation, and rising food prices, as well as short intervention lengths, contributed to null results. Our findings underscore the potential of HFVPs to improve diets and reduce nutritional disparities; however, addressing identified barriers during program design and implementation is essential to ensure that these programs achieve their goals.

**Statement of Significance**

This review provides a comprehensive synthesis of the global evidence on HFVPs, highlighting structural components that significantly influence their acceptability and effectiveness across diverse populations. By emphasizing the importance of tailored program design and examining key factors that influence success, it provides a valuable foundation for future policy development and evaluation.

## Introduction

The global prevalence of poor dietary quality is a critical public health concern, significantly contributing to noncommunicable diseases and accounting for 26% of preventable deaths worldwide [1,2]. Although regional dietary patterns vary, overall global diets remain suboptimal [2]. Diet-related deaths are linked to inadequate consumption of whole grains, fruits, and vegetables, alongside excessive intake of sodium, trans fatty acids, added sugars, processed meats, ultra-processed foods (UPFs), and sugar-sweetened beverages (SSBs) [3]. Socioeconomic status plays a key role in diet quality, with low-income populations disproportionately affected by food insecurity, leading to diets that are energy-dense but nutrient-poor [4]. Additionally, the COVID-19 pandemic, recent conflicts, and extreme weather events have worsened global diet quality and food insecurity [5,6]. Addressing this issue requires interventions that promote the consumption of nutritious options to enhance dietary quality.

Nonetheless, research worldwide has revealed that healthy, less processed foods can be significantly more expensive than highly processed, unhealthy alternatives [[7], [8], [9], [10], [11]], especially when additional costs such as time are considered. In 2021, it was estimated that >3.1 billion people globally could not afford a healthy diet [12]. Moreover, affordable UPFs high in nutrients of concern (i.e., sodium, added sugar, *trans*-fatty acids, and saturated fats) dominate the food markets in various countries [13]. Even with policies like front-of-package labels or SSBs and UPFs taxes [[14], [15], [16], [17]], people may still choose unhealthy products if healthier options are unavailable or unaffordable [18].

Healthy food incentive programs, which include discounts, matches, rebates, and voucher benefits, have emerged as a promising approach to increase dietary quality. Among these, healthy food voucher programs (HFVPs), which provide a monetary value (in the form of a voucher, token, or electronic benefits) to participants for purchasing healthy, nutrient-dense foods, are particularly noteworthy. Complementing SSBs and UPFs taxes with healthy food benefits can boost the efficacy of tax policies in improving dietary quality among low-income populations, while also easing the economic impact of the tax on them and narrowing nutritional disparities [19,20]. Some reviews suggest that healthy food incentives, including voucher benefits, help improve the purchase and consumption of healthier foods [[21], [22], [23], [24], [25], [26]]. However, limited evidence is available on the program structure components that may influence their effectiveness.

Understanding which structural features of existing HFVPs enhance participant acceptability and effectiveness in increasing consumption of nutrient-dense foods is essential for informing the design of future programs. However, available reviews that evaluate HFVPs were conducted before the COVID-19 pandemic [22,24,[26], [27], [28], [29], [30]] or have focused on peer-reviewed articles [[22], [23], [24],^26^,^30^], omitting relevant evidence contained in grey literature. Furthermore, reviews have mostly focused on the impact these programs have on nutritional indicators [22,[24], [25], [26], [27], [28], [29], [30], [31]], and less on program structure components, barriers, and enablers that could inform the design and implementation of new programs.

To address these gaps and guide policymaking on more effective implementation of these programs, this narrative review aimed to synthesize global evidence on program design, structure, and implementation components that influence the acceptability and effectiveness of HFVPs. Additionally, we summarize the outcomes of these interventions among beneficiaries.

## Methods

We conducted a narrative review of peer- and non–peer-reviewed articles describing studies that evaluated, either quantitatively or qualitatively, the program structure and/or effects of HFVPs on purchasing and consumption of healthy foods and beverages across countries and socioeconomic settings. Because the overarching objective of this review was to assess the potential of HFVPs to improve diet quality and subsequent health outcomes, we focused our framework for study identification, data extraction, and results interpretation on nutrition-related factors as described below.

Although 4 types of incentives have been identified (Table 1), this study focuses exclusively on noncash benefits providing monetary value in the form of vouchers, tokens, or debit cards to purchase healthy foods. This decision was made given the extensive history of implementation of voucher interventions, the increased global interest in implementing interventions to specifically promote healthy food purchases, and their feasibility in low- and middle-income countries (LMICs), where paper-based vouchers require less technological infrastructure than electronic systems used for other incentive types.

**TABLE 1:** Classification of food incentives aimed at increasing consumption of healthy foods^1^.

Type of food Incentive	Description
Discounts	Offers consumers a reduced price on specific healthy foods when they are purchased.
Matches	An incentive that matches all or a portion of the amount a consumer spends on eligible foods to provide additional buying power and thereby increases the amount a consumer can purchase.
Rebates	Provides cash back to a consumer after the purchase.
Voucher benefits	Provides consumers with monetary value to purchase healthy, nutrient-dense foods (e.g., fruits and vegetables) and can be delivered in the form of a voucher, token, or electronically added to a debit card.

1 Adapted from Healthy Food America [21].

Studies were identified through manual, purposive, snowball, and citation searches conducted up to March 2024. We utilized Google Scholar to identify studies through keyword combinations such as “incentive,” “subsidy,” “voucher,” “coupon,” “healthy foods,” “diet,” and “nutrition.” Policy documents and non–peer-reviewed reports were also sourced through Google searches.

Inclusion criteria included quantitative or qualitative evaluations of programs providing voucher benefits exchangeable for healthy, nutrient-dense foods with a specific goal of improving dietary outcomes among beneficiaries. Programs combining healthy food vouchers with other incentives were included if voucher benefits were reported as an independent intervention arm. Also included were programs providing unrestricted voucher benefits redeemable at local markets selling only minimally processed foods. We included only primary studies for each program, except for the United States Supplemental Nutrition Program for Women, Infants, and Children (WIC), where we included only reviews, qualitative assessments, and primary studies conducted after the 2020 revisions. Excluded were studies focusing on supply-side experiences or outcomes, and those evaluating interventions using only discounts, matches, or rebates (Table 1), which depend on consumer spending and offer heterogeneous financial incentives. In-kind food assistance and acute emergency response interventions were excluded due to challenges in valuing food items and the unique circumstances of such programs.

Similarly, produce prescriptions and school feeding programs were excluded for being venue- and population-specific, making them less comparable to community-based programs. Lastly, interventions providing voucher benefits unrelated to healthier foods or those that did not restrict products high in nutrients of concern were also excluded. For example, the United States’s Supplemental Nutrition Assistance Program (SNAP) was excluded because, at the national and state level, it did not restrict items such as SSBs at the time of our review. However, we did include evaluations of GusNIP pilots specifically targeting SNAP participants.

Based on our review’s objective and following the structure outlined by Engel and Ruder (2020) [23], we extracted and organized data according to the structural characteristics of the program as follows: participant eligibility and enrollment, delivery and timing of voucher benefit, financial value of benefits, foods eligible for voucher redemption, type of retail venue, program duration, and incorporation of nutrition education. Although program cost was of interest, insufficient information was available on this component. We also assessed the methods employed in the study and the outcomes reported. For ease of reporting, long-term interventions funded and led by governments (usually lasting >12 mo) are referred to as “programs,” whereas short-term interventions (usually lasting 12 mo or less) led by academic or nongovernmental institutions are referred to as “interventions.”

## Results and Discussion

We included 54 peer-reviewed articles, program evaluations, and reports from 7 governmental programs and 21 intervention studies from 13 countries, encompassing 6 regions (Table 2) [[32], [33], [34], [35], [36], [37], [38], [39], [40], [41], [42], [43], [44], [45], [46], [47], [48], [49], [50], [51], [52], [53], [54], [55], [56], [57], [58], [59], [60], [61], [62], [63], [64], [65], [66], [67], [68], [69], [70], [71], [72], [73], [74], [75], [76], [77], [78], [79], [80], [81], [82], [83], [84], [85]]. The following sections present the program structure components and outcomes of these HFVPs.

**TABLE 2:** Healthy food voucher programs and interventions included in the review.

Type of healthy food voucher program	Country of implementation	Program’s name or description
Governmental program	Mongolia	The Food Stamps Program (FSP) [32]
	Indonesia	Indonesia’s “Non-Cash Food Aid” program [33]
	United Kingdom (England, Wales, Northern Ireland)	The Healthy Start Program [[34], [35], [36], [37], [38], [39], [40], [41], [42]]
	Canada	The British Columbia Farmers’ Market Nutrition Coupon Program (FMNCP) [[43], [44], [45], [46]]
	South Korea	The Food Voucher Program [47,48]
	Scotland	Best Start Foods Program [49]
	United States of America	The US WIC [[50], [51], [52], [53], [54], [55], [56], [57], [58], [59]]
Intervention	United States of America, Massachusetts	Massachusetts Farmers’ Market Coupon Program [60]
	United States of America, Michigan	Project FRESH [61]
	United States of America, Utah	NA – Evaluation of Utah Farmers’ Market Incentive program [62]
	United States of America, Rhode Island	Healthy Foods, Healthy Families Initiative [63]
	United States of America, Los Angeles	NA – Intervention providing F&V benefits to WIC participants (before 2009 WIC revisions) [64,65]
	United States of America, San Francisco	NA – Intervention providing F&V supplement to WIC participants (during the COVID-19 pandemic) [66,67]
	United States of America, San Francisco	NA – intervention providing either F&V vouchers or unrestricted vouchers on a weekly or monthly basis [68,69]
	United States of America, Los Angeles and San Francisco	NA – Intervention providing F&V vouchers [70]
	United States of America, Pennsylvania	Healthy Options Project [71]
	United States of America, Wyoming	WY Markets Matter Pilot [72]
	United States of America, North Carolina	Healthy Helping [73]
	United States of America Washington D.C.	The Produce Plus Program [74]
	United Kingdom	Fresh Street Program [75,76]
	Wales	NA – Intervention providing fruit juice vouchers [77]
	France	Fruit and Vegetables at Home [78]
		NA – Intervention providing F&V vouchers [79]
	Ethiopia	NA – Intervention providing vouchers for local markets [80,81]
		United Nations’ World Food Program − Fresh Food Voucher Program [82]
	Ecuador	NA – Intervention assessing the impact of cash, vouchers, and food transfers [83]
	Cameroon	NA – Intervention providing vouchers for a standardized food basket [84]
	Haiti	The Fresh Food Voucher Project [85]

F&V, fruits and vegetables; NA, Not Applicable.

### Structure of HFVPs

**FIGURE 1:**
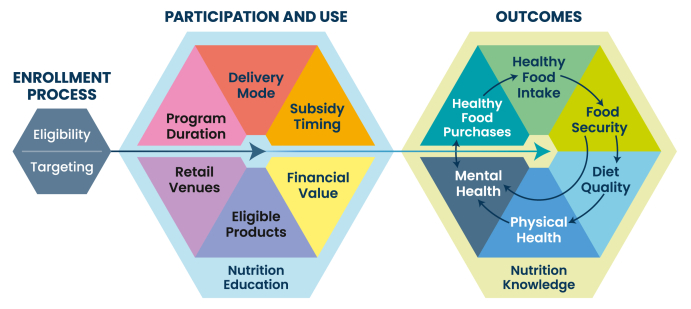
Structural components of healthy food voucher programs affecting the enrollment process, participation and use, and nutrition-related outcomes.

Focusing on the acceptability and effectiveness of HFVPs, this review identified key structural factors influencing program enrollment, participation, and impacts on nutrition-related outcomes, including healthy food purchases and intake, food security, diet quality, and physical health. As shown in Figure 1, specific factors affect the enrollment process for participants, whereas additional elements influence participation and benefit use once enrolled. Together, these factors shape the effectiveness of HFVPs in achieving their intended nutrition-related outcomes. We explore the structural factors that affect enrollment, participation, and voucher benefit use by providing the following: *1*) a brief description and main goal, *2*) common approaches, *3*) examples from high-income contries (HICs) and low- and middle-income countries (LMICs), where available, and *4*) barriers and facilitators to achieving the main goals (summarized in Table 3) [[86], [87], [88], [89], [90], [91]]. Notably, most evidence comes from HICs, as relatively few HFVPs have been implemented and evaluated in LMICs. This scarcity of programs in LMICs also explains why previous reviews have included limited evidence from these settings.

**TABLE 3:** Identified barriers and facilitators related to program structure goal that may affect acceptability and effectiveness of healthy food voucher programs in improving nutrition outcomes.

Program structure goal	Barriers	Facilitators
**Related to program enrolment**		
Effective eligibility and targeting of participants most in need	- Exclusion of individuals with uncertain immigration status despite their high need [34] - Difficulties with the “annual income” tests for families with variable earnings from self-employment [34]	- Allow community-based organizations to adjust recipient lists and appeal exclusions to improve Means Tests effectiveness is targeting low-income households [33] - Raising income thresholds in high-cost living areas to ensure those most in need are eligible [43,44,68,69,86] - Using a place-based household-level benefit to remove stigma associated with schemes which require individuals to prove need [75,76]
High enrollment of target population	- Confusing program eligibility criteria hindering participants' enrollment [34] - Lack of program awareness [34] - Complex and burdensome application process [23,37] - Language barriers [34] - Transportation costs due to in person-only enrollment options [87]	- Offering remote enrollment options to reduce transportation costs and increase accessibility and convenience [87] - Toll-free phone lines where participants can access program information and apply [88] - Single application form to apply to multiple social programs [49] - Positive interactions with program staff [50] - Offering information and assistance in multiple languages [89]
**Related to program participation and use**		
Efficient delivery and timing of benefits	- Transportation cost when required to pick up the benefit [50,51] - Delays in receiving benefits due to limited staffing [51] - Negative interactions with program staff when picking up the physical benefits [50,51] - Feelings of stigma with paper vouchers [34] - Inconvenience of having to pick up benefits on a weekly basis [68]	- Providing physical benefit in smaller amounts to facilitate transactions [45,65,69,70,72,75,76,80,85] - Implementing security features on printed vouchers to ensure their correct use [66,75,76] - Mail delivery to avoid transportation costs [67,68,70,78] - Electronic benefit renewal to avoid transportation costs [50,51] - Electronic benefits are associated with fewer feelings of stigma, less administrative burden, and improved shopping experience [50,51] - Technological barriers and network issues for using electronic benefits in low-resource rural areas [82] - Implementing hybrid delivery systems to adapt and address logistical barriers [32]
Reliable and equitable financial value of benefits	- Fixed-value benefits for large family sizes dilute purchasing power [52,69] - Inflation and rising food prices [52] - Fixed-value benefits at a national level provides a lower purchasing power in high-cost living areas [52] - Lack of efficient communication when notifying changes in benefit value [51,52]	- Subsidies adjusted to household size [47,78,79] - Allowing more than one household member to be eligible [89] - Increasing subsidy value to adjust for inflation [51,53] - Allowing benefit to roll over if not fully utilized [51]
Selection of acceptable and appropriate eligible food products	- Frozen produce vouchers can result inconvenient to participants in lower-income settings with limited facilities and equipment for food preparation [23] - The inclusion of high-cost items such as infant formula can reduce the proportion of benefit spent on other nutritious foods [34] - Having few available products suitable for people with certain allergies [50] - Overly restrictive benefits (e.g., FV-only vouchers) may be ineffective in areas lacking a variety of healthy foods [23]	- Frozen produce is appreciated in higher-income settings for its convenience [23] - Providing benefits with a wider variety of foods simplify using the full benefit amount and integrating the foods into daily meals [51] - Benefits with fewer restrictions may be considered as providing more autonomy and flexibility [72]
Accessible and appropriate retail venues	- Long distance to retailers [34,36] - Lack of market stalls offering products acceptable for culturally diverse communities [34] - Small retailers often charge higher prices [91] - Lack of advanced technology infrastructure and connectivity, distance to retail outlets, staff shortages, and limited food variety in some programs implemented in rural areas [51,82] - Negative interactions with store staff and poor customer service [34,36,51,54,78] - Lack of awareness of registered outlets or vendors [51] - In-store mislabelling of eligible products [51]	- Allowing participants to redeem their benefit in various types of retailers [34,90] - Allowing participants to redeem benefits in multiple locations [34,73] - Small retailers may be more accessible to participants compared to larger-size retailers [91] - Using Unstructured Supplementary Service Data (USSD) technology to address connectivity issues faced in rural areas [82] - Adequately trained personnel [34,36,51,54,78] - Clear and accurate labelling of eligible products [51] - Mobile application for participants to monitor their benefits and identify eligible products [52] - Allowing benefits to be used in self-service transactions or online purchases [36]
	Regarding farmers’ markets:	Regarding farmers’ markets:
	- Higher food prices, usually in HICs [46, 62,63] - Limited operating hours and inflexible work schedules [62,63] - Limited variety of foods in rural areas [45] - Physical access issues and higher transportation costs [46,62,63] - Language barriers [63]	- Improvement in the shopping experience, positive social interactions, and increased interest in local foods [44,46,62,65,71] - Enjoyable interactions with vendors (i.e., farmers) [46,51,65] - Perception of offering higher quality produce [45,60,71] - Enhancement of family connectedness and children being encouraged to consume local produce [45,46,62] - Increased confidence in preparing and cooking new types of produce [45,46]
Integration of impactful nutrition education	- Low attendance rates due to unconducive times [78] - Sessions lasting too long [80] - Information presented in a monotonous way [78]	- Engaging experiential educational activities [81] - Increased participants’ sense of belonging and expanded social networks [45]

FV, fruit and vegetables; HIC, high-income country; USSD, Unstructured Supplementary Service Data.

### Enrollment of participants and its influencing factors

#### Eligibility and targeting

Careful eligibility criteria, which define the qualifications for participation in HFVPs, and effective targeting, which involves identifying the specific groups the program aims to reach, are essential to ensure that limited resources reach those most in need. Programs typically use factors such as income and poverty level thresholds, belonging to vulnerable populations, anthropometric components, and participation in existing incentive programs to determine eligibility.

Overall, most programs use income or poverty level thresholds for eligibility [92]. A common method for social assistance is the Proxy Means Test (PMT), which estimates household income through measurable indicators like demographics and housing attributes from national household surveys [93,94]. Households scoring below a set cut-off point are deemed eligible [94]. The PMT method avoids requiring participants to report and verify their actual income, as is typically necessary with poverty level assessments, which can be burdensome for participants. This approach is widely used in LMICs [32,83]. However, the PMT faces criticism for inaccuracies and potential exclusion of eligible households [[93], [94], [95]]. To address this issue, Indonesia’s Non-Cash Food Subsidy (BPNT) program added district-specific rules and allowed local adjustments to recipient lists, enhancing the PMT’s effectiveness in targeting the lower-income households [33].

In contrast, poverty level assessments are more commonly used in HICs [71]. In the United States, federal poverty levels, issued by the Department of Health and Human Services, determine eligibility for certain programs. The United States WIC program benefits those with family income below 185% of the poverty level or who participate in other social service programs [96]. To assess the threshold criteria, this approach requires participants to report and verify their income. Moreover, interventions in high-cost living areas sometimes raise the poverty level threshold, as seen in interventions in San Francisco, United State, and British Columbia’s program, in Canada [43,44,68,69,86,97].

Income eligibility can also be coupled with criteria based on belonging to vulnerable populations [92]. For example, the Massachusetts Farmers’ Market Coupon Program provides low-income elders with coupons redeemable for fresh fruits and vegetables (FVs) [60]. Other programs and interventions offer nutritious food vouchers to low-income pregnant and postpartum women, infants, and children [34,82,89,98].

Furthermore, some interventions, both in LMICs and HICs, use targeting tools and anthropometric thresholds aimed at identifying those most in need. In France, a validated deprivation index based on an 11-item questionnaire determined eligibility for a voucher intervention [79]. In Cameroon, undernourished children, as assessed by mid-upper arm circumference, were eligible for a nutrition recovery voucher [84]. Other interventions target those already enrolled in governmental programs or nongovernmental interventions [85]. For instance, being a federal program participant has been the criterion for some interventions in the United States [[63], [64], [65], [66], [67],[72], [73], [74]].

#### Barriers and facilitators

Eligibility criteria should be carefully designed to guarantee that those with the greatest needs are not excluded. Although most identified barriers to ensuring equitable eligibility criteria come from programs in HICs, they may also be relevant to LMIC settings. As noted in Table 3, eligibility-related issues include confusing criteria tied to tax credits and concerns about excluding vulnerable groups like asylum seekers [34,35]. Additionally, requiring proof of need can result in increased stigma [75,76], which has led interventions to use a community eligibility approach [75,76]. Further research is needed to assess the impact of such interventions on participation and diet quality.

#### Enrollment process

Once eligibility criteria have been thoughtfully defined, achieving high enrollment of eligible participants is essential for HFVPs to have a positive impact on health and nutrition. Various enrollment processes have been used, including both in-person and remote options.

In-person enrollment options typically require participants to visit the program’s office to verify the required documentation. In the United States’s WIC program, individuals can contact local agencies to set up appointments, often through a toll-free number [88]. They are informed about the nearest location and the required documentation to determine eligibility [88]. Financial constraints may limit agencies’ capacity to accommodate all eligible individuals, leading to waiting lists and a priority system to manage benefits distribution [88]. Moreover, healthcare providers can note if a patient could be eligible for WIC and make referrals to the WIC clinic [99]. Other options include relying on community partners to enroll low-income households [86], enrollment processes at clinics [66], and having sign-up tables at the farmers’ markets where vouchers can be used, informing potential beneficiaries through multi-language flyers at health clinics, community centers, and neighborhoods [63]. In LMICs, community health workers or nongovernment organization staff are commonly responsible for the enrollment of participants [84,85].

Remote enrollment options have also been used in HICs to reduce transportation costs and increase accessibility [89]. In the UK’s Healthy Start Program, participants can now apply online, by email, or by phone, providing specific information and documentation. Accepted applicants receive their cards by mail [34]. Moreover, in response to the COVID-19 pandemic, nearly all WIC local agencies shifted their enrollment process and conducted it remotely, making it safer, more accessible, and more convenient for participants’ schedules [87]. As a result, a combination of in-person and remote enrollment options has become a common practice [73].

#### Barriers and facilitators

Enrollment processes can present barriers that may significantly affect program participation (Table 3). For example, in England, a decline in program uptake was attributed to several factors, including the requirement for a health professional to sign an application before submission, low understanding of the program, and insufficient program awareness [[34], [35], [36], [37]]. Additional challenges include transportation costs related to in-person enrollment and complex application processes [34,36], impacting effectiveness and acceptability [23,37]. Although these barriers have been reported in HICs, they may also apply to LMICs.

Facilitators to enrollment include user-friendly application processes and having remote options [49]. Furthermore, positive interactions between program staff and applicants encourage ongoing engagement [50]. In response to various evaluations, the UK Healthy Start program simplified its application process in April 2020 to increase enrollment and uptake [37]. Although it is uncertain if these approaches would be effective in LMICs, the principles of streamlining processes and reducing application time are likely to be beneficial.

### Factors affecting program use and participation

#### Delivery mode and timing of benefits

The efficient delivery and timing of benefits can significantly influence participant engagement in the program. Key considerations when defining the benefit distribution method include selecting the appropriate mode of delivery and frequency.

Voucher benefits can be distributed physically or electronically, each with distinct advantages and challenges. Physical forms include paper vouchers, stamps, tickets, or tokens, often delivered as a lump sum but sometimes in smaller amounts to facilitate transactions [45,65,69,70,72,75,76,80,85]. This mode is commonly implemented in both HICs and LMICs. Some interventions have implemented multiple security features on printed vouchers to avoid their misuse [66,75,76]. Physical benefits are usually collected at designated locations such as agency offices, healthcare facilities, grocery stores, or farmers’ markets [74,80,81,84,97]. To reduce transportation costs, some programs and interventions use mail delivery [67,68,70,78].

Electronic distribution, such as prepaid cards, offers a modern alternative. The United States WIC program, UK Healthy Start, Scotland’s Best Start Foods, and Indonesia’s food voucher programs provide participants with a prepaid card in which funds are added periodically [33,89,90,100]. An intervention in Ethiopia piloted digital vouchers via SMS, using a point-of-sale system enabled by Unstructured Supplementary Service Data (USSD) technology [82]. However, there is currently no evidence on the use of prepaid cards in LMICs, and further research is needed to determine whether similar approaches would work in these settings.

The timing of benefit distribution also varies, with most programs and interventions offering benefits monthly, although some use weekly or biweekly schedules [64,68,77,84,97]. A San Francisco intervention tested weekly compared with monthly voucher delivery, finding lower use rates with weekly distribution [68], suggesting that weekly frequency might be inconvenient for participants [68].

#### Barriers and facilitators

In low-income settings, reported challenges include technological barriers, network issues, and a lack of interest among some beneficiaries and vendors [82]. These barriers are often more prevalent in rural areas than in urban ones. To address logistical barriers, a hybrid delivery system in Mongolia used paper stamps in rural areas and electronic cards in urban areas [32].

Electronic benefits have the potential to increase program use, as they tend to be more acceptable to beneficiaries. They can enhance the shopping experience by reducing delays, negative interactions with program staff, and the stigma associated with paper vouchers [49,50,51,101].

### Financial value of benefits

A reliable and equitable financial value of benefits is essential to encourage enrolled participants to use them. The value of benefits in HFVPs varies widely, complicating comparisons across programs. Factors like currency depreciation and purchasing power of benefits further affect comparisons, especially across countries. For instance, a 200 Ethiopian Birr voucher benefit (∼$10 USD) in Ethiopia represented ∼15% of a household’s monthly expenditure [80,81], whereas a $40 USD voucher benefit in Ecuador was estimated to be roughly 10% [83]. Additionally, benefits have ranged from as low as $5 USD [60] to as high as $80 USD per month across interventions [72], with most interventions, both in LMICs and HICs, usually providing $40 USD per month or less.

An important distinction can be made between programs that provide fixed benefits [50,65,[67], [68], [69], [70],^73^,[80], [81], [82], [83], [84], [85]] and those that adjust the benefit to other variables, such as household size, which has been identified as a factor that might improve the program’s impact. Various HFVPs do adjust benefit value to the household size [47,78,79] or household composition using specific formulas (e.g., South Korea’s Food Voucher program and British Columbia’s Farmers' Market Nutrition Coupon Program (Canada) [43,44,47]. Alternatively, other programs allow more than one household member to be eligible with different values based on age groups [32,36,49,96].

Some programs have increased the amount of the benefits to address inflation, particularly since 2020, such as the UK Healthy Start program and Indonesia’s BPNT program [33,37,90]. The United States WIC program also responded to the COVID-19 pandemic economic crisis by temporarily increasing FV benefits from $9–$11 USD to $35 USD monthly, later adjusting to $24–$47 USD [67,102].

#### Barriers and facilitators

Inflation and rising food prices pose challenges to the benefits impact, undermining voucher benefits for nutrient-dense foods [52]. To mitigate this, the United States’s WIC program offers benefits redeemable for specific quantities of food, helping families maintain their purchasing power for most eligible food items, irrespective of retail prices [52]. However, for the fixed cash-value benefit allotment for FV in the WIC food package, participants receive a set monetary amount rather than a fixed-unit benefit [52]. Before the changes that occurred during the COVID-19 pandemic, participants in the United States WIC and UK Healthy Start programs revealed that this FV benefit amount was inadequate, as the quantity of foods that it could purchase decreased over time [35,36,[50], [51], [52]].

Effective HFVPs require careful consideration of benefit value and family needs, especially amidst inflation and rising food prices. Increasing benefit size periodically to account for inflation enhances participants’ acceptability of and participation in the program and may improve the program’s outcomes. Evaluations of the WIC program after the benefit increases in 2020 showed improved food security, benefit redemption [53], and positive participant perceptions [51].

### Eligible products

The range of food products eligible for voucher benefits varies widely across programs, influenced by target population needs and local market availability. Eligible products must be both nutrient-dense and acceptable to participants to ensure they are purchased and consumed. Key themes include the focus on specific food categories, local availability, program scale, and participants’ resources and preferences.

Some interventions focus exclusively on FV, including fresh, canned, frozen varieties, and 100% fruit juices [63,67,68,69,70,75,78,79]. Other interventions limit vouchers to fresh produce only or specific products like 100% fruit juice [60,63,74,75,77,79]. Different programs specify eligible foods based on local availability and intervention scale. In Ethiopia, vouchers were exchangeable for items such as cabbage, tomato, mango, orange, carrot, onions, and eggs [82], and Indonesia's BPNT program included eggs in the rice voucher initiative to enhance dietary diversity [33].

Additionally, some interventions allow a broader range of nutrient-rich foods [33,43,44,47,46,49,81,83,90,103]. In 2020, Indonesia’s BPNT program evolved into the Sembako program, expanding eligible items to include various local grains, eggs, beef, chicken, fish, vegetable proteins, as well as FV [33]. The UK Healthy Start and Scotland’s Best Start Foods programs cover fresh, frozen, and canned FV; cow milk; fresh, dried, and canned pulses; fresh eggs, and infant formula [49,90].

Certain programs also aim to boost local agricultural demand. South Korea’s Food Voucher Program restricts benefits to domestic agricultural products, including fresh produce, grains, milk, and tofu [47,48], whereas Haiti’s intervention offered separate vouchers for fresh and staple foods [85]. Moreover, the United States WIC program has evolved over the years to include additional nutrient-dense foods and is periodically updated to reflect the Dietary Guidelines for Americans [103].

#### Barriers and facilitators

Designing the optimal food basket requires considering participants’ facilities and preferences.

Restrictive benefits (e.g., FV-only vouchers) can be less acceptable for some participants [36,72], and researchers caution that they could increase food insecurity in areas lacking diverse healthy foods, as their access would be limited [23]. Ensuring that programs accommodate diverse dietary needs and local market conditions is also crucial for their success and participant engagement [51]. However, reported barriers include inflexibility concerning allergies, cultural preferences, and individual tastes [50].

### Participating retail venues

The selection of accessible and appropriate retail venues is a crucial factor in the success and acceptability of HFVPs. Key components in this area include the location of retail venues and their associated challenges, the amount and type of venues included in the program, and the role of adequately trained personnel.

Well-functioning food markets are essential for the successful implementation of HFVPs [84,104]. Most of the short- and medium-term interventions, both at HICs and LMICs, have been concentrated in urban areas due to logistical challenges in rural regions. For instance, in Haiti and Ecuador, food vouchers could be exchanged at retailers located in urban districts [83,85]. In the United States, the Healthy Helping program allowed redemption at any of the nearly 500 locations of a supermarket chain within North Carolina [73].

Moreover, the Healthy Start program in England, Wales, and Northern Ireland allows card usage in multiple venues, including supermarkets, convenience stores, drug stores, markets, news dealers, freezer and locker meat provisioners, and petrol stations [90]. Evaluations have indicated that ∼30% of the benefits were redeemed in retail outlets other than large supermarkets [34]. In the United States WIC program, authorization may be granted to large grocers, supermarkets, supercenters, medium-sized grocers, or small retailers such as convenience stores and pharmacies to participate as vendors [105].

Indonesia’s BPNT program allowed voucher use at small shops registered as remote agents for the state-owned bank, chosen to implement the program in various districts. These shops received debit card readers from the bank for direct transactions [33]. Moreover, with the goal of increasing the resilience of the local food system, the Fresh Street intervention in the UK allowed participants to redeem their vouchers only at locally owned FV shops [75,76].

#### Barriers and facilitators

Including supermarkets in HFVPs has made it easier for some participants to use benefits, as these stores are part of their regular shopping routines [35]. To enhance access and reduce distance barriers, several programs have partnered with various types of retail venues. The inclusion of medium, small, and non-traditional retailers is important for participants who do not live near large stores [91]. However, it is also important to consider administrative costs when expanding these venues. Understanding and prioritizing locations where beneficiaries primarily shop is essential for maximizing the effectiveness of program resources.

Rural programs, both in HICs and LMICs, face barriers such as a lack of advanced technology infrastructure and connectivity, distance to retail outlets, staff shortages, and limited food variety [51,78,82]. Nevertheless, some interventions, such as the World Food Programme’s initiative in Ethiopia’s rural markets, have made strides. This intervention used digital vouchers via the USSD system, benefiting both participants and vendors [82].

Additionally, the success of interventions relies not only on the strategic selection of retail venues but also on the participants’ experiences within these venues. Well-trained store personnel play a crucial role in this context. Negative interactions with store staff, especially cashiers, have been a significant barrier [34,36,51,54,78]. Other barriers include a lack of awareness of registered outlets or vendors, and embarrassment at checkout due to in-store mislabeling of eligible products [34,50,51,54].

Clear and accurate labeling at the point of selection is crucial for participants’ store preferences and voucher use [51]. Moreover, random observations and secret shoppers have been used in program evaluations to ensure proper cashier acceptance of vouchers [70,76]. Addressing these barriers is essential to ensure participant engagement and high redemption rates.

### Incorporation of farmers’ markets

Farmers’ markets, which refer to local marketplaces where farmers and producers sell their goods directly to consumers, have become strategic venues for promoting nutrient-dense foods and addressing food insecurity, especially in HICs [44]. Various HFVPs now incorporate these markets to offer participants more options for redeeming benefits.

Some interventions allow participants to use benefits across various retail types, including farmers’ markets [68, [64], [65], [66], [67],^78^], whereas others restrict benefits use exclusively to farmers’ markets [43,60,[61], [62], [63],^71^,^72^,^74^].

Several benefits associated with using farmers’ markets for HFVPs have been identified, including reducing food insecurity, supporting the local economy, and improving access to nutritious foods [45,46,62,63,72,106]. For instance, in a FV-voucher intervention in Los Angeles, participants using supermarkets increased fresh produce consumption by 0.8 servings per 1000 kcal compared with baseline consumption, whereas those attending farmers’ markets saw a 1.4 servings increase [65].

#### Barriers and facilitators

Farmers’ markets positively affect food-shopping practices, social interactions, and interest in local foods [44,46,62,65,71]. These markets provide unique social environments where participants enjoy interactions with farmers and vendors [46,51,65]. Shopping with children at these markets also enhances family connectedness and encourages children to try, prepare, and consume local produce [45,46,62].

However, barriers to shopping at farmers’ markets exist. In HICs, food prices at these markets are usually higher than in other venues [46,62,63]. Limited operating hours and inflexible work schedules also pose challenges [62,63]. Some programs at farmers’ markets are only available during certain months of the year, further restricting access [74]. In rural areas, both in HICs and LMICs, the variety of available foods can be limited [45]. Physical access issues and higher transportation costs than grocery stores are also concerns [46,62,63].

### Duration of interventions

Besides the ongoing governmental programs, the duration of HFVPs varies widely, but most interventions are short-term, typically lasting 6 months or less [[43], [44], [45], [46],[61], [62], [63],[65], [66], [67], [68], [69],[70], [71], [72], [73], [74],[80], [81], [82], [83], [84], [85]]. Many interventions lasted between 1 and 4 months [[43], [44], [45], [46],^62^,^63^,^67^,[71], [72], [73],[80], [81], [82],^84^,^85^]. These brief durations are common across various regions and intervention designs. Although some interventions extend slightly longer, they often remain under a year. Examples include the Fresh Street UK intervention [75,76], which spanned between 4 to 11 months, and 2 interventions in France, which lasted 12 months [79,78,107].

Researchers have noted that the short duration of many of these interventions poses challenges for assessing long-term impacts and sustainability. The temporary nature of financial support often leads to changes in participants’ experiences during the intervention, but these benefits can diminish once the intervention ends, causing increased financial stress and a return to less nutritious food purchases [46]. Moreover, the lack of follow-up periods in many studies prevents the evaluation of long-term trends and the effectiveness of benefits, raising questions about the sustainability of positive outcomes post-intervention [24]. Longer-duration studies and strong evaluations of ongoing programs are needed to track health outcomes beyond behavioral changes and ensure HFVPs’ lasting benefits [22].

### Integration of nutrition education

Nutrition education has been integrated into HFVPs through varied topics, facilitators, and methods, with the goal of engaging participants, encouraging behavioral change and strengthening the program’s impact. Although some interventions offer education as an optional component [44,45,63], others require attendance to receive benefits [83].

In HICs, sessions often cover the importance of increasing FV consumption [38,[77], [78], [79]]; healthy eating for disease prevention, meal planning, and including milk, vitamins, and fresh foods in the diet [38]. In LMICs, they focus on dietary diversity [82], maternal and child feeding practices [83,81], benefits of vitamin A–rich FV and animal-source foods [80], hygiene and food safety [84,85], and the consequences of malnutrition [80]. The personnel delivering these sessions also differs: dietitians, physicians, and midwives commonly lead in HIC [71,[77], [78], [79]], whereas community health workers often facilitate sessions in LMICs [81,84]. Venues include health facilities [38,80], community centers [78], home visits [84], and farmers’ markets [63].

To foster behavioral change, some interventions focus solely on theoretical advice, including distribution of posters and flyers, leaflets, healthy FV-based recipes, or dietary advice through text messages [38,48,77,79,83]. Other interventions use experiential learning methods such as cooking workshops and food demonstrations [44,63,78,80,81,84,85], role-playing [81], gardening classes and farm visits [71], and individualized home visits [84].

Evidence suggests that combining food benefits with experiential nutrition education is most effective [108]. In Ethiopia, combining food vouchers with nutrition education significantly improved child feeding practices and reduced undernutrition, compared to either intervention alone [81]. Similarly, in British Columbia (Canada), incorporating nutrition skill-building activities resulted in improvements in healthy eating practices and reported increases in beneficiaries' sense of belonging and expanded social networks [45].

#### Barriers and facilitators

Overall, integrating nutrition education enhances nutrition knowledge and encourages healthier diets in both HICs and LMICs [61,[77], [78], [79],^80^,^81^,[83], [84], [85]]. Success, however, depends on the structure and delivery of sessions, as well as attendance. Active learning strategies such as food demonstrations help maintain engagement and improve outcomes [80,61]. Requiring participation can also ensure positive outcomes, as in Ecuador, where food vouchers with mandatory nutrition education activities increased dietary diversity [83].

However, low attendance can undermine effectiveness. In France, no association was found between workshop attendance and FV consumption, possibly due to only 50% participation [78]. Prolonged sessions in Ethiopia also led to decreased attendance [80]. Therefore, designing engaging activities and scheduling them at convenient times is crucial for ensuring high attendance and achieving positive outcomes.

### Outcomes

HFVPs have been evaluated for their impact on a range of nutritional outcomes. The Supplemental Material outlines some of the common assessment methods used in these evaluations. In this section, we discuss the reported effects of HFVPs on several key outcomes, including the purchase and consumption of healthy foods, food security, diet quality, physical health indicators, and mental health.

#### Changes in healthy food purchase and consumption

Evidence from various countries indicates that HFVPs can increase the purchase and consumption of subsidized items (Table 4). This increase has been observed in multiple interventions focused solely on FV vouchers [52,[63], [64], [65], [66],[70], [71], [72],^75^,[77], [78], [79],^82^]. However, the choice of products may hinge on factors like availability, seasonality, affordability, and low perishability. For example, in an Ethiopian intervention, despite increased FV purchases, the most commonly purchased items were bananas, onions, and potatoes [82].

**TABLE 4:** Healthy food voucher programs’ outcomes based on the evaluated studies.

Outcomes evaluated	Number of resources reporting this outcome (% out of all resources)	Characteristics of results	Results^1^	Overall interpretation of the evaluated studies^2^
**Healthy food purchase**	21 (39%)	Number (%) of articles quantitatively reporting positive effects, location, and references	*n* (%): 10 (48%) Locations: Pennsylvania, United States; LA, United States; North Carolina, United States; Overall United States; Ethiopia; United Kingdom References: [40,[56], [57], [58],^64^,^71^,^73^,[80], [81], [82]] 3	Most evaluated evidence on HFVPs demonstrates that they may be effective in increasing the purchase of eligible healthy foods.
		Number (%) of articles qualitatively reporting positive effects, location, and references	*n* (%): 10 (48%) Locations: England; UK; United States; British Columbia (Canada); Scottland References: [34,36,42,45,49,50,52,55,75,76]	
		Number of articles reporting null results, location, and references	*n* (%): 1 (5%) Location: UK Reference: [37]	
**Healthy food consumption**	34 (63%)	Number (%) of articles quantitatively reporting positive effects, location, and references	*n* (%): 22 (65%) Locations: Pennsylvanya, United States; LA, United States; San Francisco, United States; Rhode Island, United States; Wyoming, United States; Michigan, United States; Overall, United States; England; Ethiopia; France; Indonesia; Wales; South Korea References: [33,38,39,47,48,53,[56], [57], [58],^61^,^63^,^65^,^66^,^69^,[70], [71], [72],^75^,[77], [78], [79],^81^] 3	The effectiveness of HFVPs in increasing the consumption of eligible healthy foods has shown mixed results. However, the majority of evaluated studies indicate that participants have increased their consumption of healthy foods. Reported reasons for null results include lack of transportation and equipment for food storage and preparation.
		Number (%) of articles qualitatively reporting positive effects, location, and references	*n* (%): 10 (16%) Locations: France; England; British Columbia (Canada); United States; Haiti; Utah, United States; Scottland References: [34,46,49,[50], [51], [52],^55^,^62^,^78^,^85^]	
		Number of articles reporting null results, location, and references	*n* (%): 6 (18%) Locations: France; San Francisco, United States; California, United States; Overall United States; UK References: [41,68,69,67,78]	
**Diet quality indicators**	13 (24%)	Number (%) of articles quantitatively reporting positive effects, location, and references	*n* (%): 8 (62%) Locations: Ethiopia; Mongolia; Ecuador; South Korea; LA and San Francisco; United States; Overall United States References: [32,47,56,57,70,80,83,86] 3	The effectiveness of HFVPs in increasing diet quality indicators has shown mixed results in the evaluated evidence. Reported reasons for null results include insufficient amount of the benefit to generate a robust effect, inflation and rising food prices, and short intervention length.
		Number (%) of articles qualitatively reporting positive effects, location, and references	*n* (%): 2 (15%) Locations: Hati; United States References: [55,85]	
		Number of articles reporting null results, location, and references	*n* (%): 5 (38%) Locations: San Francisco, United States; Overall United States; British Columbia (Canada); Ethiopia References: [43,56,59,68,80] 3	
**Food security**	10 (19%)	Number (%) of articles quantitatively reporting positive effects, location, and references	*n* (%): 8 (80%) Locations: Mongolia; Ecuador; British Columbia (Canada); Ethiopia; Wyoming, United States; San Francisco, United States; Overall, United States References: [32,44,53,66,68,72,80,83]	Most evaluated evidence on HFVPs demonstrates that they are effective in increasing the food security of participants.
		Number (%) of articles qualitatively reporting positive effects, location, and references	*n* (%): 1 (10%) Location: Scotland Reference: [49]	
		Number of articles reporting null results, location, and references	*n* (%): 2 (20%) Locations: Mongolia; California, United States References: [32,67]	
**Nutrition knowledge**	5 (9%)	Number (%) of articles quantitatively reporting positive effects, location, and references	*n* (%): 3 (60%) Locations: Ethiopia; Cameroon References: [80,81,84]	All evidence on HFVPs combined with nutrition education demonstrates that they are effective in increasing nutrition knowledge.
		Number (%) of articles qualitatively reporting positive effects, location, and references	*n* (%): 2 (40%) Locations: British Columbia (Canada); Haiti References: [46,85]	
		Number of articles reporting null results, location, and references	*n* (%): 0 (0%)	
**Physical health indicators**	6 (11%)	Number (%) of articles quantitatively reporting positive effects, location, and references	*n* (%): 5 (83%) Locations: Ethiopia; Wales; Cameroon; San Francisco, United States; Overall, United States References: [57,66,76,77,84] 3	The effectiveness of HFVPs in improving physical health indicators has shown mixed results in the reviewed evidence, varying based on the specific indicator being evaluated.
		Number (%) of articles qualitatively reporting positive effects, location, and references	*n* (%): 0 (0%)	
		Number of articles reporting null results, location, and references	*n* (%): 2 (33%) Locations: France; Overall, United States References: [57,79]∗^3^	
**Mental health**	4 (7%)	Number (%) of articles quantitatively reporting positive effects, location, and references	*n* (%): 0 (%)	The effectiveness of HFVPs in improving mental health has shown mixed results in the evaluated evidence, with positive outcomes reported in qualitative evaluations.
		Number (%) of articles qualitatively reporting positive effects, location, and references	*n* (%): 3 (75%) Locations: British Columbia (Canada); Scotland References: [45,46,49]	
		Number of articles reporting null results, location, and references	*n* (%): 1 (25%) Location: British Columbia (Canada) Reference: [44]	

Percentages are based on articles including the specific outcome listed.

HFVP, healthy food voucher program; LA, Los Angeles; UK, United Kingdom; WIC, The Supplemental Nutrition Program for Women, Infants, and Children.

1 Studies may be in the “Positive Impact Observed” cell and “Null Results” cell if positive results were found in one indicator or subgroup of the population studied and null results were observed in another indicator or subgroup (e.g., adults vs. children).

2 Interpretation is based on the evaluated studies. No formal quality assessment was conducted, so these statements should be interpreted with caution.

3 Includes review studies of the United States WIC program.

Broader voucher benefit interventions targeting various healthy foods also showed positive outcomes [33,36,38,39,40,43,45,47,49,[55], [56], [57], [58],^73^]. The United States WIC program has been associated with increased purchasing of healthy products and reduced purchasing of less healthy foods and beverages [57], with these effects persisting even after participants leave the program [56,58].

However, some studies report minimal or no significant associations in the purchase or consumption of subsidized foods [37,41,53,68, 69,79]. For example, although the WIC program has shown improvements in healthy food consumption, the increase in fruit and vegetable intake has been minimal [57,58]. Additionally, mixed results have been observed in the UK’s Healthy Start program [37,38]. Price inflation during the study period might have diminished the benefit’s impact. Researchers suggest that additional resources, such as transportation support, cooking skills, kitchen equipment, or engaging nutrition education, might be necessary to enhance healthy food intake, especially for those with lower baseline healthy food consumption [69].

#### Changes in food security

Most studies examining the effect of HFVPs on food security consistently demonstrate a positive impact in both HICs and LMICs, with all of them evaluating this indicator at a household level [32,44,49,68,53,56,66,72,81]. Even programs with no significant dietary quality improvements showed positive impacts on food security [53]. For instance, British Columbia’s FMNCP did not improve diet quality indicators, but sustained reductions in marginal household food insecurity were observed even 16 wk post-interventions [44]. Researchers suggest that improvements in diet quality may not be achievable until households are food secure [44].

#### Changes in dietary quality indicators

HFVPs can improve diet quality, although the impact varies by context and population. Several programs and interventions have demonstrated positive effects on dietary quality [[32], [33], [34],^38^,^47^,^56^,^57^,^70^,^80^,^81^,[83], [85]]. For instance, HEI improvements have been observed in the South Korea program participants [47] and infants and children participating in the United States WIC program [56]. Improvements in dietary diversity, which is a proxy for dietary quality, have been observed in HFVPs implemented in Ethiopia, Mongolia, and Ecuador [32,83,81].

However, not all programs achieved significant dietary quality improvements [43,46,68]. Although WIC has been associated with better diet quality during pregnancy, a study using repeated, cross-sectional national survey data found no significant improvements in HEI scores among WIC mothers [59]. The impact of Healthy Start vouchers on diet quality in the UK was mixed in some assessments, with some women using the vouchers to save money rather than improve their diet [42,73].

The limited or no impact of some HFVPs on dietary quality may be attributed to several factors. Small sample sizes [56] and the short duration of voucher benefit interventions may pose obstacles to detecting substantial changes in diet quality [43,46]. Additionally, insufficient benefit amounts and high food costs may hinder participants from improving their dietary quality [43].

#### Changes in nutrition knowledge

Nutrition knowledge has been enhanced in programs incorporating behavioral change communication components [46,81,85]. Additionally, nutrition knowledge and engagement in nutrition education shape food purchasing and consumption behaviors. An intervention conducted in France revealed that participants with limited initial nutrition knowledge, who did not attend nutrition workshops, tended to use vouchers for familiar products and allocated saved funds toward other items, including sugary snacks and fast food [78]. Conversely, those with limited initial nutrition knowledge who participated in workshops increased their intake of FV, aligning with workshop recommendations [78].

#### Changes in physical health and mental health indicators

The limited studies assessing the effects of HFVPs on physical health outcomes have primarily focused on children, undernutrition, and micronutrient indicators, yielding mixed results. Reduced stunting and considerable improvement in the height-for-age distribution were observed in children of parents receiving both vouchers and nutrition education in Ethiopia [81]. Cameroon’s fresh food voucher intervention significantly improved mid-upper arm circumference in children, with a high undernutrition recovery rate [84]. In a fruit juice voucher intervention, serum β-carotene concentration significantly increased in the voucher group [77]. Moreover, WIC participation during pregnancy is associated with longer gestations and higher birth weights [56,57]. Conversely, some interventions did not show significant changes. For example, a FV voucher intervention showed no differences in vitamin status after 3 mo despite increased consumption in France [79].

Mental health outcomes have rarely been assessed. In British Columbia’s FMNCP, quantitative and qualitative evaluations of mental health outcomes showed mixed results. Quantitative assessments found no significant differences in mental well-being scores between intervention and control groups [44]. Researchers suggest factors such as stress due to persistent food insecurity may contribute to this finding [44]. Nonetheless, qualitative assessments indicated positive changes in dietary intake and beneficial effects on physical and mental health [46]. Participants reported that shopping at farmers’ markets alleviated feelings of isolation and anxiety due to social interaction [45,46].

### Program and study limitations, gaps, and opportunities

Several limitations affect the overall effectiveness of HFVPs and the generalizability of their findings. A significant limitation in interventions is their typically short duration, often lasting only a few weeks to several months, with most studies lacking follow-up periods [22,24,43]. This limits the evaluation of the benefits’ long-term trends and effectiveness. Additionally, researchers evaluating long-term governmental programs face challenges due to the absence of optimal control groups [35,56,73].

Another critical limitation is the limited amount of some benefits provided, especially when they are fixed amounts, which may not lead to significant improvements in diet quality or sustain those improvements over time [43,68]. The lack of formative research and minimal process evaluations in many studies also raises concerns; without these evaluations, it is challenging to determine whether interventions failed due to inherent flaws or improper implementation [22].

The limited scope of some studies, focusing only on subsidized foods without considering overall food purchases and dietary intake, restricts understanding of potential substitution and compensation effects [22,24]. Furthermore, small and convenience samples in many interventions hinder the generalizability of the results, making it difficult to apply findings to broader populations [24]. Lastly, the absence of cost-effectiveness analyses prevents comparisons across program design scenarios, limiting the identification of the most cost-efficient interventions [24].

To address these limitations, future research should focus on key areas. Examining underlying contextual factors, such as intervention location, can provide insights into how different environments influence effectiveness [43]. Conducting dose-response analyses to determine whether the impact of benefits varies with the amount received or spent is also crucial [43]. Statistically powered samples and evaluations of intervention responses across subpopulations are needed [25].

Longer-duration studies are necessary to track behavioral changes and health outcomes over time [22]. Future interventions should rely on solid formative research, include detailed reporting, and incorporate thorough process evaluations to assess implementation fidelity [22]. Investigating substitution and compensation effects of pricing interventions is essential for understanding broader impacts on dietary behavior [22]. Consistent data collection methods across studies would facilitate better comparisons and conclusions [43]. Finally, although not covered in this review, identifying and evaluating policies to enhance the effective involvement of the supply side in HFVPs is essential for ensuring healthy food availability to participants.

Although these recommendations highlight critical areas for future research, it is also important to acknowledge the limitations of this review, which may influence the scope and interpretation of the findings. One limitation is that the review was restricted to English-language sources, which may underrepresent research from non–English-speaking contexts; although articles were screened in both English and Spanish, no Spanish-language studies met our criteria. Additionally, although our findings may be relevant to programs using various types of healthy food incentives, our review specifically focused on programs providing healthy food voucher benefits. Further research should explore whether additional facilitators or barriers to uptake exist for other types of healthy food incentives and whether the type of incentive influences program impact on health and nutrition-related outcomes. Finally, we did not perform a formal quality assessment of the studies, meaning that findings from both robust and less rigorous designs are included. To address this, we prioritized synthesizing key insights rather than drawing definitive conclusions about effectiveness. Despite these limitations, this review provides valuable perspectives on the structure and implementation of HFVPs.

## Conclusion

Healthy food voucher programs may be a promising strategy for improving dietary quality, food security, and nutrition-related outcomes among diverse populations. This narrative review comprehensively examines the literature on programs using voucher benefits for healthy foods, highlighting their structural components and summarizing effects on food purchasing and consumption, diet quality, and other key outcomes. Evidence suggests that healthy food benefits may effectively increase the purchase and consumption of subsidized items, yielding positive outcomes in various countries, including improvements in food security indicators and enhanced nutrition knowledge among participants. However, factors such as the use of remote enrollment options, provision of electronic benefits, adequate benefit amounts, inclusion of diverse eligible healthy foods, and integration of nutrition education may affect the ability of these programs to achieve positive nutrition-related outcomes. Policymakers should account for these complex, intersecting factors when designing HFVPs to effectively address the unique needs and preferences of diverse populations.

## Author contributions

The authors’ responsibilities were as follows – JLA, CC, SWN, LST: designed the research and methodology; JLA: conducted the research, analyzed data, wrote the manuscript; all authors: critically reviewed and edited the manuscript, have responsibility for the final content, and read and approved the final manuscript.

## Funding

Supported by Bloomberg Philanthropies, Healthy Food Policy Program, grant number 2019-71181. Additional support was received by Fondecyt Regular (CC, #1240833).

## Conflict of interest

The authors report no conflicts of interest.
