# Supplementary material for: Healthy Food Voucher Programs: Global Evidence on Structure, Implementation, and Nutrition-Related Outcomes
Source: Adv Nutr. 2025 Oct 1;16(11):100530. doi: 10.1016/j.advnut.2025.100530 (PMC12589872; doi:10.1016/j.advnut.2025.100530)
Supplement: multimedia component 1 [file mmc1.docx]

# **Supplementary Material**

## **Outcome Assessment Methods**

#### Data on Food Purchases

The impact of healthy food subsidies on purchasing behaviors is often evaluated by examining changes in food purchases. Some program evaluations measure both food purchases and consumption to assess diet quality, while others use purchase data as a proxy for consumption (1, 2). A key distinction is that purchases data reflect household choices, whereas consumption data may reflect either individual-level or household-level choices.

Common tools for collecting expenditure data include questionnaires and surveys, which gather self-reported information on household spending at retail venues, types of food purchased, and non-food expenditures (3-7). For instance, in Rhode Island, USA, pre- and post-intervention surveys inquired about the proportion of the family’s budget spent on FV (4). Some programs have asked vendors to track coupon purchases using checkboxes on coupons or have used voucher logs, aiding in the monitoring of purchases, distribution, and redemption (5, 8, 9).

Another method for the collection of food purchases data is the use of electronically recorded, supermarket-obtained point-of-sale (POS) transactions (2, 6), considered a more objective measure compared to self-reports. The WIC program collects monthly food benefit redemption data to evaluate changes in household FV purchases (10). In South Korea, POS data allows analysis of all products purchased using food vouchers, including non-food items (6, 11).

Assessing purchases from various stores is crucial since recipients often use personal funds for additional food purchases, affecting overall dietary intake (12). Studies have highlighted the need to evaluate purchases of both healthy and unhealthy food categories to understand the true impact of subsidies (2, 12). Furthermore, technologies like USSD have been used in low-income settings, such as in rural Ethiopia, to collect detailed purchasing data via SMS, providing real-time information on itemized food prices and quantities without extra costs to retailers or recipients (13).

#### Data on Food Consumption

Assessing food consumption is essential for evaluating dietary quality and the impact of programs on consumption patterns at individual or household levels. Individual-level intake assessment tools include the 24-hour dietary recall (24HR), which captures detailed information on all foods and beverages consumed by a participant in the past 24 hours (3, 12, 14-17). Multiple recalls are typically conducted to account for daily variations in diet (14, 15). Qualified interviewers are typically needed for the administration of 24 HR; however, automated self-administered tools are also available. Such is the case of the Automated Self-Administered 24-Hour Dietary Assessment Tool (ASA24) used in British Columbia’s FMNCP (16).

Intake assessment tools that can evaluate either individuals or households include the food frequency questionnaires (FFQs), dietary screeners, and qualitative dietary questionnaires. FFQs are widely used, listing foods and beverages with response options for usual consumption frequency (18-21). FFQs can be self-administered, require less literacy, may include portion size information (22). Dietary screeners assess the consumption of specific food groups (9, 23), such as the USA National Cancer Institute’s 16-item FV Screener, which measures the frequency and portion size of FV consumed over the past month (9). Qualitative dietary questionnaires tailored to program objectives also provide valuable insights (4, 20, 24). For example, a French intervention asked participants if their FV consumption increased over 12 months and why (20). Similarly, a Rhode Island intervention asked if families ate more fresh FV due to the program (4).

Various methods are employed to collect consumption data, including in-person interviews, online questionnaires, and telephone interviews, often provided in multiple languages to avoid barriers (12, 14-16, 25). Moreover, given the existing food and beverage variations between and within countries, adaptation and validation of assessment tools to the local context have been made across programs and interventions (10, 16, 18, 19).

The selection of dietary intake assessment tools should be done based on the program’s objective, outcome to measure, participants conditions, and available resources. Both 24HR and FFQ usually require 30 to 60 minutes to complete (22, 26). To reduce participant burden, monetary incentives are often offered (5, 16). Moreover, given that the commonly used dietary assessment tools are subject to recall and social desirability biases, programs and interventions have opted to complement these tools with food purchase data.

#### Additional Assessment Methods and Indicators

Programs often evaluate additional outcomes such as dietary quality, food security, nutrition knowledge, and physical health indicators.

To assess dietary quality, various indicators are used, depending on the unit of measurement. At the individual level, both in adults and children, indicators used include the Minimum Dietary Diversity, Minimum Acceptable Diet, Minimum Meal Frequency, Children Dietary Diversity Score, Healthy Eating Index, and the Alternative Healthy Eating Index (6, 12, 16, 17, 27, 28). At the household level, the Household Dietary Diversity Score has been more commonly used (7). Information on how to construct each of the indicators, their uses, strengths and weaknesses have been extensively published (29).

Food security is commonly assessed at a household level using tools like the USDA six-item Food Security Survey Module, which has high sensitivity and specificity and is validated in English and Spanish (2, 9, 10, 12, 14, 23, 30). In Canada, a modified version of the Household Food Security Survey Module was used (5). Other used indicators include the Food Consumption Score and household hunger scale (7, 27, 28, 31). Models assessing food security as a continuous variable have also been used (23).

Interventions with a nutritional education component often assess changes in nutrition knowledge using Knowledge, Attitudes, and Practices (KAP) surveys, a widely accepted tool for investigating health-related behaviors (32, 33). Similarly, parental nutrition knowledge was evaluated in Ethiopia using a survey module that included over 25 questions on topics covered during the nutrition education program (27).

Physical health indicators have also been assessed to a lesser extent (see Table 4 of the main paper). These have included vitamin C and beta-carotene status, blood pressure, blood glucose, triglycerides, cholesterol, weight, height, waist and hip circumference, and body composition (15, 19). Indicators like Body Mass Index for adults and length-for-age and weight-for-height for children have been derived from these measures (15, 27). In low-income settings, middle-upper arm circumference has also been assessed in children (15, 27). Moreover, malnutrition risk was evaluated using the Malnutrition Universal Screening Tool (MUST) in Canada(5).

Interest in the mental health effects of HFSP is growing. The FMNCP in Canada, for example, measured mental well-being using the Warwick-Edinburgh Mental Well-Being Scale, which assesses positive affect and psychological functioning (5). Furthermore, qualitative assessments, such as focus group discussions and structured interviews, have been used to gather participants’ perceptions of program components. These methods explore advantages and disadvantages, application process experiences, subsidy usage, and perceived dietary behavior changes (34-38). Tools like the Consolidated Framework for Implementation Research and the Theoretical Framework of Nutritious Food Access have been used to guide the development of qualitative assessment instruments (38-40).

**References**

1. Parnham J, Millett C, Chang K, Laverty AA, Von Hinke S, Pearson-Stuttard J, et al. Is the healthy start scheme associated with increased food expenditure in low-income families with young children in the United Kingdom? BMC Public Health. 2021;21(1).

2. Lowery CM, Henderson R, Curran N, Hoeffler S, De Marco M, Ng SW. Grocery Purchase Changes Were Associated With A North Carolina COVID-19 Food Assistance Incentive Program. Health Affairs. 2022;41(11):1616-25.

3. Herman DR, Harrison GG, Jenks E. Choices Made by Low-Income Women Provided with an Economic Supplement for Fresh Fruit and Vegetable Purchase. Journal of the American Dietetic Association. 2006;106(5):740-4.

4. Bowling AB, Moretti M, Ringelheim K, Tran A, Davison K. Healthy Foods, Healthy Families: combining incentives and exposure interventions at urban farmers’ markets to improve nutrition among recipients of US federal food assistance. Health Promot Perspect. 2016;6(1):10-6.

5. Aktary ML, Dunn S, Sajobi T, O’Hara H, Leblanc P, McCormack GR, et al. The British Columbia Farmers’ Market Nutrition Coupon Program Reduces Short-Term Household Food Insecurity Among Adults With Low Incomes: A Pragmatic Randomized Controlled Trial. Journal of the Academy of Nutrition and Dietetics. 2023.

6. Kim YJ, Kim S. Food and Nutrition Assistance Policies in Korea: Focus on Food Voucher Program. FFTC Agricultural Policy Platform. 2023.

7. Hidrobo M, Hoddinott J, Peterman A, Margolies A, Moreira V. Cash, food, or vouchers? Evidence from a randomized experiment in northern Ecuador. Journal of Development Economics. 2014(107):144-56.

8. Relton C, Crowder M, Blake M, Strong M. Fresh street: the development and feasibility of a place-based, subsidy for fresh fruit and vegetables. Journal of Public Health. 2022;44(1):184-91.

9. Porter CM, Wechsler AM, Naschold F. WY Markets Matter Pilot Study Results: Farmers Market Coupons Improve Food Security and Vegetable Consumption. Journal of Hunger & Environmental Nutrition. 2022;17(1):126-34.

10. Whaley SE, Anderson CE, Tsai MM, Yepez CE, Ritchie LD, Au LE. Increased WIC Benefits for Fruits and Vegetables Increases Food Security and Satisfaction Among California Households with Young Children. Journal of the Academy of Nutrition and Dietetics. 2023.

11. Ji JH, Park HJ, Lee SH. Behavioral Interventions to Improve Dietary Quality for Low-Income Households: A Field Experiment in South Korea. 2023 Annual Meeting, July 23-25, Washington DC; Washington D.C.: Agricultural and Applied Economics Association; 2023.

12. Basu S, Gardner CD, White JS, Rigdon J, Carroll MM, Akers M, et al. Effects Of Alternative Food Voucher Delivery Strategies On Nutrition Among Low-Income Adults. Health Affairs. 2019;38(4):577-84.

13. Dad F, Dibari F, Kebede A, Lefu E, Ndumiyana T, Butaumocho B. Digitalisation in the WFP fresh food voucher programme: a pilot study from rural Amhara region, Ethiopia. Frontiers in Nutrition. 2023;10.

14. Herman DR, Harrison GG, Afifi AA, Jenks E. Effect of a Targeted Subsidy on Intake of Fruits and Vegetables Among Low-Income Women in the Special Supplemental Nutrition Program for Women, Infants, and Children. American Journal of Public Health. 2008;98(1):98-105.

15. Teta I, Foudjo BUS, Nielsen JN, Oben J, Nguefack-Tsague G, Ntentie FR, et al. Outcomes of a food voucher program and factors associated with the recovery rate of children with moderate acute malnutrition in Far North Cameroon. J Health Popul Nutr. 2023;42(1):37.

16. Aktary ML, Dunn S, Sajobi T, O'Hara H, Leblanc P, McCormack GR, et al. Impact of a farmers’ market healthy food subsidy on the diet quality of adults with low incomes in British Columbia, Canada: a pragmatic randomized controlled trial. The American Journal of Clinical Nutrition. 2023;117(4):766-76.

17. Basu S, Akers M, Berkowitz SA, Josey K, Schillinger D, Seligman H. Comparison of Fruit and Vegetable Intake Among Urban Low-Income US Adults Receiving a Produce Voucher in 2 Cities. JAMA Network Open. 2021;4(3):e211757-e.

18. Mouratidou T, Ford FA, Wademan SE, Fraser RB. Are the benefits of the ‘Healthy Start’ food support scheme sustained at three months postpartum? Results from the Sheffield ‘before and after’ study. Maternal &amp; Child Nutrition. 2010;6(4):347-57.

19. Bihan H, Méjean C, Castetbon K, Faure H, Ducros V, Sedeaud A, et al. Impact of fruit and vegetable vouchers and dietary advice on fruit and vegetable intake in a low-income population. European Journal of Clinical Nutrition. 2012;66(3):369-75.

20. Buscail C, Margat A, Petit S, Gendreau J, Daval P, Lombrail P, et al. Fruits and vegetables at home (FLAM): a randomized controlled trial of the impact of fruits and vegetables vouchers in children from low-income families in an urban district of France. BMC Public Health. 2018;18(1).

21. Ford FA, Fraser RB, Mouratidou T, Wademan SE. Effect of the introduction of ‘Healthy Start’ on dietary behaviour during and after pregnancy: early results from the ‘before and after’ Sheffield study. British Journal of Nutrition. 2008;101(12):1828-36.

22. National Cancer Institute. Food Frequency Questionnaire at a Glance: The Dietary Assessment Primer Website; [Available from: <https://dietassessmentprimer.cancer.gov/profiles/questionnaire/>.

23. Ridberg RA, Marpadga S, Akers MM, Bell JF, Seligman HK. Fruit and Vegetable Vouchers in Pregnancy: Preliminary Impact on Diet & Food Security. Journal of Hunger & Environmental Nutrition. 2021;16(2):149-63.

24. Anderson JV, Bybee DI, Brown RM, McLean DF, Garcia EM, Breer ML, et al. 5 A Day Fruit and Vegetable Intervention Improves Consumption in a Low Income Population. Journal of the American Dietetic Association. 2001;101(2):195-202.

25. Burr M, Trembeth J, Jones K, Geen J, Lynch L, Roberts Z. The effects of dietary advice and vouchers on the intake of fruit and fruit juice by pregnant women in a deprived area: a controlled trial. Public Health Nutrition. 2007;10(6):559-65.

26. National Cancer Institute. 24-hour Dietary Recall (24HR) at a Glance The Dietary Assessment Primer Website [Available from: <https://dietassessmentprimer.cancer.gov/profiles/recall/>.

27. Han Y, Park S, Kim J, Hoddinott J. Engaging Fathers Through Nutrition Behavior Communication Change Does Not Increase Child Dietary Diversity in a Cluster Randomized Control Trial in Rural Ethiopia. The Journal of Nutrition. 2023;153(2):569-78.

28. Han Y, Kim HB, Park S. The Roles of Nutrition Education and Food Vouchers in Improving Child Nutrition: Evidence from a Field Experiment in Ethiopia. Journal of Health Economics. 2021;80.

29. International Dietary Data Expansion Project. Data4Diets: Food Security Indicators [Available from: <https://inddex.nutrition.tufts.edu/data4diets/indicators>.

30. Ridberg RA, Levi R, Marpadga S, Akers M, Tancredi DJ, Seligman HK. Additional Fruit and Vegetable Vouchers for Pregnant WIC Clients: An Equity-Focused Strategy to Improve Food Security and Diet Quality. Nutrients. 2022;14(11):2328.

31. MacAuslan I, Attah R. Food stamps for food security: the impact of a targeted social assistance programme in Mongolia. Journal of Poverty and Social Justice. 2015;23(2):121-34.

32. Action Contre La Faim - Haiti. Fresh Food Vouchers to Strengthen Diet Diversification and Improve Resilience. Action Contre La Faim; 2012.

33. Andrade C, Menon V, Ameen S, Kumar Praharaj S. Designing and Conducting Knowledge, Attitude, and Practice Surveys in Psychiatry: Practical Guidance. Indian Journal of Psychological Medicine. 2020;42(5):478-81.

34. McFadden A, Green JM, Williams V, McLeish J, McCormick F, Fox-Rushby J, et al. Can food vouchers improve nutrition and reduce health inequalities in low-income mothers and young children: a multi-method evaluation of the experiences of beneficiaries and practitioners of the Healthy Start programme in England. BMC Public Health. 2014;14(1):148.

35. Bertmann FMW, Barroso C, Ohri-Vachaspati P, Hampl JS, Sell K, Wharton CM. Women, Infants, and Children Cash Value Voucher (CVV) Use in Arizona: A Qualitative Exploration of Barriers and Strategies Related to Fruit and Vegetable Purchases. Journal of Nutrition Education and Behavior. 2014;46(3):S53-S8.

36. Duffy EW, Vest DA, Davis CR, Hall MG, De Marco M, Ng SW, et al. “I Think That’s the Most Beneficial Change That WIC Has Made in a Really Long Time”: Perceptions and Awareness of an Increase in the WIC Cash Value Benefit. International Journal of Environmental Research and Public Health. 2022;19(14):8671.

37. Martinez CE, Ritchie LD, Lee DL, Tsai MM, Anderson CE, Whaley SE. California WIC Participants Report Favorable Impacts of the COVID-Related Increase to the WIC Cash Value Benefit. International Journal of Environmental Research and Public Health. 2022;19(17):10604.

38. Caron-Roy S, Dunn S, Elliott C, Fournier B, Lashewicz B, Leblanc P, et al. ‘My coupons are like gold’: experiences and perceived outcomes of low-income adults participating in the British Columbia Farmers’ Market Nutrition Coupon Program. Public Health Nutrition. 2022;25(2):410-21.

39. Gago CM, Wynne JO, Moore MJ, Cantu-Aldana A, Vercammen K, Zatz LY, et al. Caregiver Perspectives on Underutilization of WIC: A Qualitative Study. Pediatrics. 2022;149(2).

40. Caron-Roy S, Lee YY, Sayed SA, Lashewicz B, Milaney K, Dunn S, et al. Experiences and Perceived Outcomes of Low-Income Adults During and After Participating in the British Columbia Farmers’ Market Nutrition Coupon Program: A Longitudinal Qualitative Study. Journal of the Academy of Nutrition and Dietetics. 2022;122(12):2257-66.
